# Ultra-Wideband RCS Reduction Based on Non-Planar Coding Diffusive Metasurface

**DOI:** 10.3390/ma13214773

**Published:** 2020-10-26

**Authors:** Guozhang Wu, Wenqi Yu, Tao Lin, Yangyang Deng, Jianguo Liu

**Affiliations:** 1 State Key Laboratory on Integrated Optoelectronics, Institute of Semiconductors, Chinese Academy of Sciences, Beijing 100083, China; wgzhang16@semi.ac.cn (G.W.); yuwenqi16@mails.ucas.ac.cn (W.Y.); 2 College of Materials Science and Opto-Electronic Technology, University of Chinese Academy of Sciences, Beijing 100049, China; dengyangyang18@mails.ucas.ac.cn; 3 College of Information and Navigation, Air Force Engineering University, Xi’an 710077, China; ltzhineng@semi.ac.cn; 4 School of Microelectronics, University of Chinese Academy of Sciences, Beijing 100029, China

**Keywords:** non-planar, radar cross-section (RCS), ultra-wideband, discrete particle swarm algorithm, metasurface

## Abstract

A novel non-planar coding metasurface optimized by discrete particle swarm algorithm (DPSO) is proposed in terms of the property of wideband radar cross-section (RCS) and diffuse scattering. The design consists of two unit cells, “0” and “1”, which have a 180° ± 37° phase difference for phase interference cancellation. The 10 dB monostatic RCS reduction frequency range of the metasurface is from 6.4 to 29.6 GHz, and its bandwidth ratio is 4.62:1, under normal incidence of the two polarizations. Compared to the planar surface, the non-planar surface has a greater bandwidth with respect to the monostatic and bistatic RCS reduction. The results declare its properties of ultra-wideband, angle insensitivity, and polarization insensitivity. Finally, the theoretical analysis, simulation, and experimental results match perfectly, indicating that the metasurface can be used in the RCS reduction or other microwave applications with wider RCS reduction and diffuse scattering.

## 1. Introduction

With the rapid development of modern microwave radio frequency and detection technology, it is urgent to efficiently combat system detection and tracking. As an important physical quantity to describe the electromagnetic (EM) scattering of objects, radar cross-section (RCS) has received extensive attention and should be reduced particularly. At present, the most widely considered RCS reduction methods can be sorted into two categories, radar-absorbing metamaterials (RAMs) technique and phase interference cancellation technology [1]. The incident EM waves can be absorbed and converted into heat based on the RAMs technology, which is feasible to control the RCS of the targets [2,3,4,5]. Traditional microwave absorbers reported are usually in the form of the Salisbury screens or Jaumann screens, but they are affected by their thick sizes [6,7,8]. Meanwhile, limited absorption bandwidth is the main problem that they can hardly meet the demand of broadband RCS reduction [9,10]. Furthermore, the energy absorbed from the EM waves is more likely to be captured by infrared detectors, thus increasing the possibility of exposing the targets.

The phase interference cancellation technology originates from the classic 180° phase-difference between the corresponding reflections. The most typical case is employing the combination of Artificial Magnetic Conductor (AMC) and Perfect Electric Conductor (PEC) to generate RCS reduction by transferring the energy from the normal direction to other directions [11]. Although some excellent results are obtained, its bandwidth is limited by the AMC bandwidth. To overcome this drawback, a checkerboard arrangement structure, using AMC technology, is applied to realize broadband RCS reduction, and its 10 dB monostatic RCS reduction bandwidth takes up 40% of the total operating frequency [12]. These periodic rectangular checkerboard metasurfaces generate four scattering beams, and their bistatic RCS reduction is about 8.1 dB [11,12,13,14]. Besides, in Reference [15], C. A. Balanis et al. propose a hexagonal checkerboard periodic structure, which can reflect the incident wave into six beams, and the 10 dB monostatic RCS reduction bandwidth reaches about 61%. The checkerboard structure has limited ability to further improve bandwidth, and its control methods are not flexible enough.

In recent years, coding metasurface has been used to obtain broadband RCS reduction, which performs more accurately than checkerboard structures in controlling EM waves [16,17]. It can be traced back to Sievenpiper, who applies frequency-selective surfaces for phase control [18]. The concept of coding metamaterials was proposed by Cui et al. [19], in 2014, and experimentally verified. They designed two units with phase response of “0” and “π” to simulate the digital states of “0” and “1”, and the EM waves can be controlled by designing coding sequences [20,21]. The RCS reduction of the coding diffusion metasurface can be further decreased based on the optimization algorithm [22]. For larger RCS reduction, Su et al. propose a metasurface with random 3-bit coding metamaterials with a 10 dB monostatic RCS reduction ranging from 7.9 to 20.8 GHz [23]. A metasurface based on the hybrid array pattern synthesis and particle swarm optimization (PSO) is designed by Su et al. [24] and achieves 80.2% bandwidth, covering from 15.6 to 36.5 GHz. Furthermore, Moccia introduces a novel suboptimal design strategy with a negligible calculation amount, whose result is comparable to the typical method based on brute force numerical optimization, thus paving the way for structural design with arbitrarily large electrical dimensions [25,26]. The metasurfaces mentioned above are all based on planar structures; however, the non-planar structure is more feasible to achieve greater bandwidth than the planar structure. Liu et al. select two kinds of Minkowski fractal structures, to create an uneven metasurface and realize an RCS reduction more than 10 dB from 5.8 to 18.0 GHz, with a bandwidth of 105.2% [27]. Su et al. proposed a novel uneven-layered coding metamaterial and obtained a bandwidth more than 120% [28]. However, they do not consider the influence of polarization and angle on the structure.

In this paper, a novel non-planar coding metamaterial based on two different circle ring models is proposed, to create an ultra-wideband RCS reduction metasurface, and it shows the property of perfect polarization and angle insensitivity in the simulation and experiment. The main contributions of this paper are as follows:

- The non-planar structure introduces a variable parameter of height, compared with the planar structure, so that more basic meta-particles with phase differences are greatly added, increasing the ability to manipulate EM waves. The 10 dB RCS reduction frequency range of the metasurface varies from 6.4 to 29.6 GHz (129% bandwidth) and is greater than that of planar structures.
- The metasurface presents a great polarization and angle insensitivity when the EM waves work under wide oblique incidence degrees in both simulation and experiment. The reason is that the circle ring structure of the unit cells has better stability due to its isotropic, when compared to other structures.
- An efficiently discrete particle swarm algorithm (DPSO) algorithm that evaluates each parameter in a discrete pattern containing only 0 and 1 in each iteration is employed to optimize the metasurface instead of the traditional PSO algorithm. The optimal metasurface has a greater bandwidth of the monostatic RCS reduction, compared to the checkerboard surface, and divides the incident EM waves into more energy side lobes than four beams of the checkerboard surface.

## 2. Unit Cell Design

**Figure 1:**
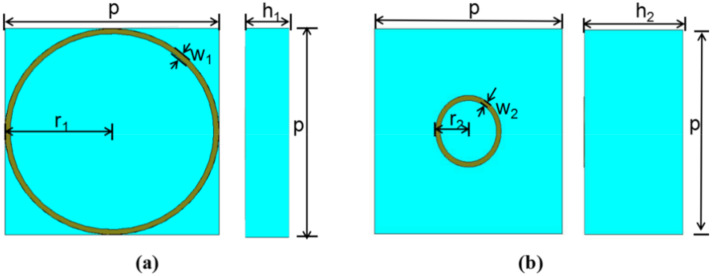
The model and geometry of the unit cells: (**a**) the top view and side view of the unit cell 1, and (**b**) the top view and side view of the unit cell 2.

**Figure 2:**
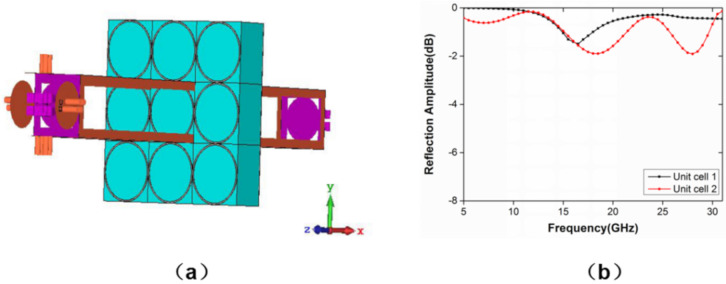
(**a**) The unit cell simulation model based on the Floquet method and (**b**) reflection amplitude of the unit cells.

The ring structure is not sensitive to the incident angle and polarization, which shows perfect stability. Meanwhile, the circular ring structure has better isotropic than the square ring structure [28], which is conducive to further stability of the structure. Figure 1 shows the top view and side view of the 3D model with the symmetrical structure that includes the circle ring model. The unit cell is printed on the substrate with a thickness of *h* and dielectric constant of *ε_r_* = 4.3 (loss tangent *tan δ* = 0.022). A Frequency Domain Simulation based on the Finite Element Analysis with periodic boundary conditions (PBC) is employed to simulate the reflection amplitude and the phase of the model. The simulation is carried out in the form of a Floquet port shown in Figure 2a, where the boundary conditions are set as the unit cells in the x- and y-directions, and a Floquet port is set in the z-direction. This unit cell can be modeled as an infinite periodic structure. Through a series of optimization, two unit cells with different side length, *L,* and thickness *h* are set as two basic super cells. The periodic *p* is 8 mm, and the ring width *w_1_* and *w_2_* are both 0.2 mm. The amplitude in Figure 2b is somewhat lower than average intensity because of absorption at resonance frequencies, which can be easily understood using the equivalent circuit method [29]. However, absorption is much weaker than reflection and generally cannot be comparable with the reflection for both unit cells.

The RCS reduction of the metasurface, compared with an equal-sized PEC, can be expressed by the following [15]:

(1) $$\mathrm{RCS}\;reduction=10\mathrm{lg}\frac{{\left|E^{s}\right|}^{2}}{{\left|E^{i}\right|}^{2}}$$

Then, the checkerboard metasurface of two unit cells can conclude the RCS reduction by the following:

(2) $$\mathrm{RCS}\;reduction=10\mathrm{lg}{\left|\frac{A_{1}e^{j\varphi_{1}}+A_{2}e^{j\varphi_{2}}}{2}\right|}^{2}$$

where *A_1_* and *A_2_* are the reflection amplitudes, and *φ_1_* and *φ_2_* are the reflection phases of two unit cells, respectively. For infinite metasurface, the reflection amplitudes *A_1_* and *A_2_* can be equal to each other. Therefore, Equation (2) can be simplified as follows:

(3) $$\mathrm{RCS}\;reduction=20\mathrm{lg}\left|\frac{e^{j(\varphi_{1}-\varphi_{2})}}{2}\right|$$

**Figure 3:**
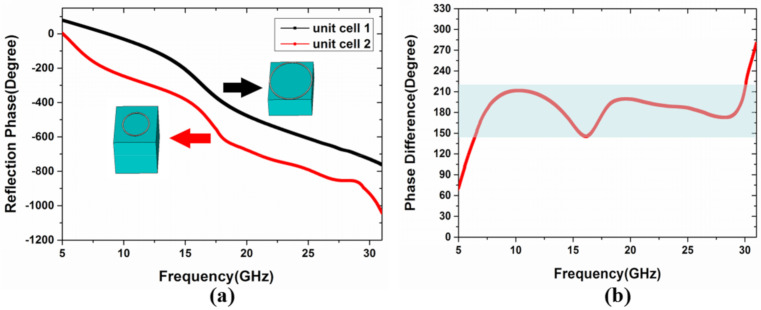
(**a**) The reflection phase and (**b**) the phase difference of the unit cells.

From Equation (3), it can be figured that, to get 10 dB RCS reduction, the phase difference between the unit cells should vary from 143° to 217°, to satisfy 180° ± 37° phase difference. Then, a simple optimization algorithm can be employed to deal with the simulation results, and the unit structures are selected corresponding to the maximum bandwidth. The radius *r_1_* and the thickness *h_1_* of the unit cell 1 are 4 and 3 mm, respectively. The radius *r_2_* and the thickness *h_2_* of the unit cell 2 are 1.4 and 6 mm, respectively. The calculation results are shown in Figure 3. Firstly, Figure 3a shows the reflection phases of two unit cells in the frequency range from 6.4 to 30.2 GHz. Then, based on the above results, the phase difference of 180° ± 37° can be realized and shown in Figure 3b. Compared with previously reported results [12,13,14,15,16,17,22,23,24], the non-planar circle ring structure can obtain a larger bandwidth, which is beneficial to the preparation of broadband metasurfaces. It is worth mentioning that the physical mechanism of a non-planar structure is introducing a variable parameter of height, compared with a planar structure, so that basic meta-particles with variable phase differences are greatly added and increase the ability to manipulating the EM waves. It is important for basic meta-particles to make more free combinations to realize super-wideband phase cancellation.

**Figure 4:**
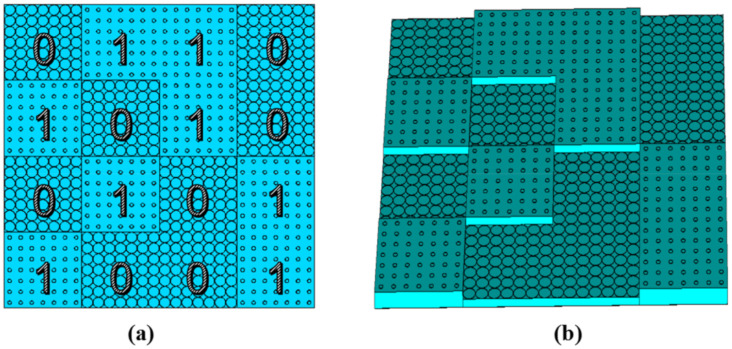
The (**a**) two-dimensional geometry structure and (**b**) three-dimensional geometry structure.

The ratio of center wavelength 16.4 mm (18.3 GHz) to the unit cell period 8 mm is 2.05 under normal incidence. The depth of the coding metasurface is 3 mm for the height of the unit cells 3 and 6 mm. Thus, the ratio of the depth to wavelength varies from 0.06 to 0.3 in the frequency range from 6 to 30 GHz, and the ratio to the center wavelength is 0.18, which is a sub-wavelength scale. To meet the periodic boundary conditions of the unit cell, a lattice consists of 7 × 7 unit cells, and the whole metasurface with 4 × 4 lattices is proposed in Figure 4. The overall dimension of the metasurface is 224 mm × 224 mm. Based on the design of digital elements, the metasurface is composed of various coding sequences, which contains 1-bit codes, “1” and “0”. The two codes indicate different reflection phases that “1” element represents 180° phase response and “0” represents 0° phase response, with 180° phase differences. In our design, elements “0” and “1” correspond to unit cell 1 and unit cell 2, respectively.

Here, a DPSO algorithm is employed, to optimize the metasurface. In the PSO algorithm, every particle can be regarded as an individual that finds the best solution through cooperation in individuals and shares information, which is an intelligent optimization algorithm proposed by Kenndey and Eberhart [30,31]. The DPSO algorithm in the letter is a little different from traditional PSO that evolutes in a continuous particle speed and population location, but it evaluates each parameter in a discrete pattern, such as only containing 0 and 1 in each iteration. It is more efficient and faster than the traditional PSO algorithm. Then, the DPSO algorithm with array theory is used to optimize the layout distribution of the metasurface, to obtain the optimal coding sequences.

For a metasurface constituting *M × N* lattices, these lattices’ intervals *dx* and *dy* are in the *x*- and *y*- direction, respectively. The scattering model of the metasurface at angle *θ* and *φ* is described by the following:

(4)  E(θ,φ)=EP(θ,φ)⋅AF(θ,φ)

where *θ* and *φ* are used to denote elevation and azimuth angles of an arbitrary scattering direction. EP and AF are the element pattern function of a lattice and the array factor, respectively. Here, we regard EP as a constant. According to the array theory, the array factor can be expressed by the following:

(5) $$AF\left(\theta ,\varphi \right)=\sum_{i}^{M}\sum_{j}^{N}\exp\{-j[2\pi \sin\theta \left(\cos\varphi \cdot id_{x}+\sin\varphi \cdot jd_{y}\right)/\lambda +\phi (i,j)]\}$$

where $\phi \left(i,j\right)$ denotes the phase of the elements, including 0° and 180°. The possible solutions in DPSO algorithm can be considered as a particle, and the mathematical description of the speed and position of the next generation can be expressed as follows:

(6) $$\nu_{m}^{n}=w*\nu_{m}^{n}+c_{1}*rand(0,1)*(pbest_{m}^{n}-x_{m}^{n})+c_{2}*rand(0,1)*(gbest^{n}-x_{m}^{n})$$

where *pbest^n^_m_* is the individual optimal location, and *gbest^n^_m_* is the global optimal location. The function rand (0,1) indicates a random number chosen from a uniform distribution between 0 and 1; *c_1_* and *c_2_* are two acceleration constants (often chosen to be 2.0 from experience); and *w* is the weight factor (often chosen to be 0.6) [24]. The DPSO module evaluates the fitness, updates the particle speed and the population location (i.e., the phase arrangements) in each iteration, and then sends the information to the evaluation module.

(7) fitness=maxAF(θ,φ)

**Figure 5:**
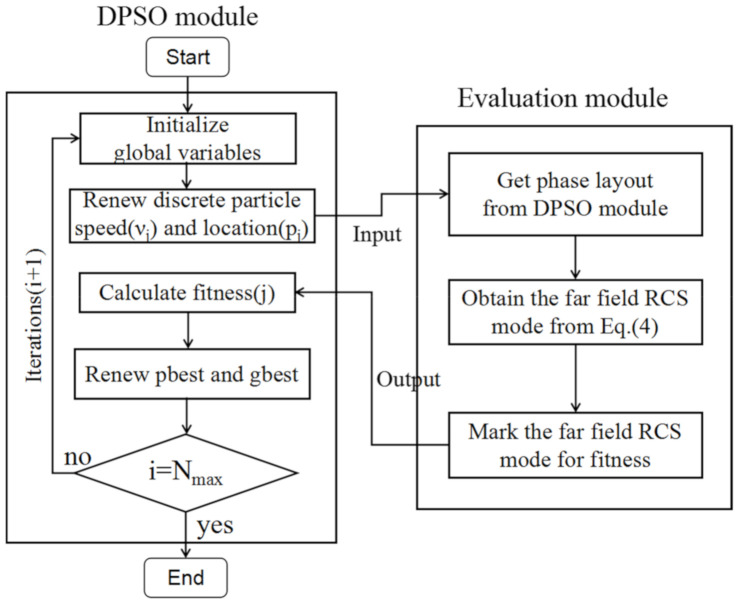
The flowchart of discrete particle swarm algorithm (DPSO) module and Evaluation module.

The maximum value of the scattered field is employed as the fitness function, to manipulate the scattered wave, and the fitness function must be minimized by using an algorithm that generates a random set of binary sequences. The following DPSO module and Evaluation module are carried out for the element optimization in Figure 5. The latter gives an optimized metasurface scattering pattern based on the above Evaluation module and calculates the maximum RCS and the monostatic RCS. Then mark the fitness and return to the DPSO module. After a series of iterations, the best phase layout of the metasurface with the lowest RCS can be obtained in Figure 4a.

This paper indicates an improved DPSO algorithm, which is characterized by the introduction of height variables, to obtain more complex coding units for its benefit, to satisfy the opposite phase condition under broadband conditions. We want to stress that it is characterized by the broadband effect in this paper, instead of algorithm innovation, compared with Moccia [25,26]. The DPSO algorithm is similar to a genetic algorithm and evolutionary algorithm in some aspects, but requires less computational storage and usually only a few lines of code. Thus, the DPSO algorithm is proposed to optimize the layout of the metasurface. Additionally, it is possible to employ FPGA, digital metamaterial, and the hardware to generate the bits 0 and 1 for the field-programmable in this letter, such as loading pin diodes to coding metasurface [21,32], to generate the opposite phases, or utilizing an electronically reconfigurable reflectarray to dynamically modulate the phase by wave front shaping [33].

## 3. Simulation and Analysis

**Figure 6:**
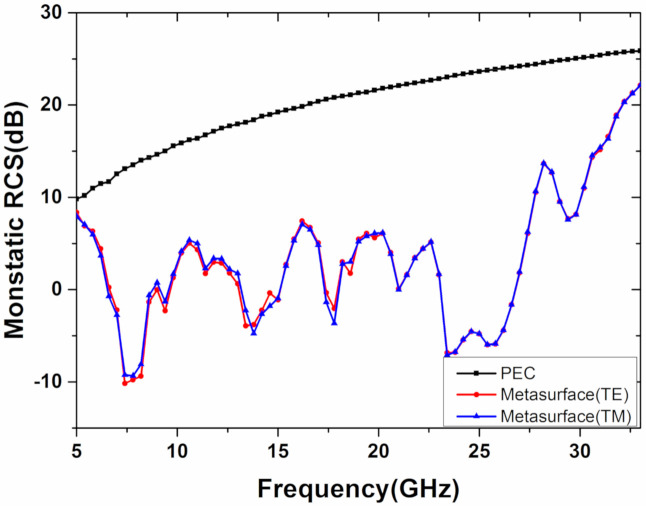
The simulation of the monostatic radar cross-section (RCS) between the metasurface and the equal-sized Perfect Electric Conductor (PEC) under TE and TM wave.

**Figure 7:**
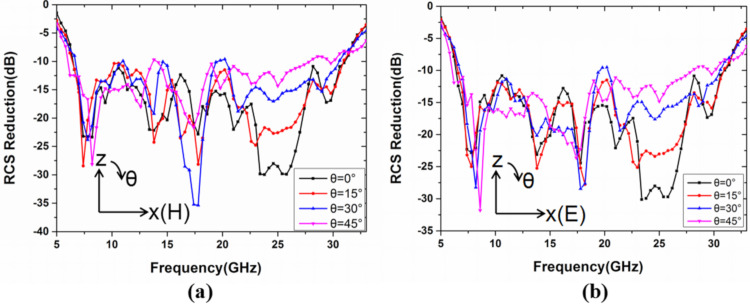
The simulation of the bistatic RCS reduction for the metasurface, under different incident angles, including 0°, 15°, 30°, and 45°, in (**a**) TE wave and (**b**) TM wave.

To confirm the broadband RCS reduction and diffuse scattering effect for the proposed metasurface, a Time Domain Simulation based on the Finite Integration Method is employed to simulate Full-wave simulation of the metasurface. In Figure 6, the monostatic RCS reduction of the metasurface is more than 10 dB from 6.4 to 31 GHz (132% bandwidth), under normal incident, compared with the equal-sized PEC surface. It is worth noting that the simulation result is consistent with the bandwidth range covered by the phase difference of the two units in Figure 3 (frequency range from 6.4 to 30.2 GHz with 130% bandwidth). Furthermore, the RCS reduction under normal incident, in two different polarizations, are simulated, and the results are highly consistent in both 0° and 90° polarization, which proves polarization-insensitive. Simultaneously, the RCS reduction of the metasurface under different incident angles in an *x*–*z* plane for simplicity is also carried out to simulate, including 0°, 15°, 30°, and 45°, under TE and TM waves, as shown in Figure 7. As the incident angle increases, the RCS reduction of the metasurface in the TE and TM modes remains more than 10 dB in the frequency range from 6.4 to 31 GHz. It indicates that the structure is not only polarization-insensitive but also not affected by the incident angle below 45°, and the results are consistent with our original intention of choosing the circle ring structure for isotropic.

**Figure 8:**
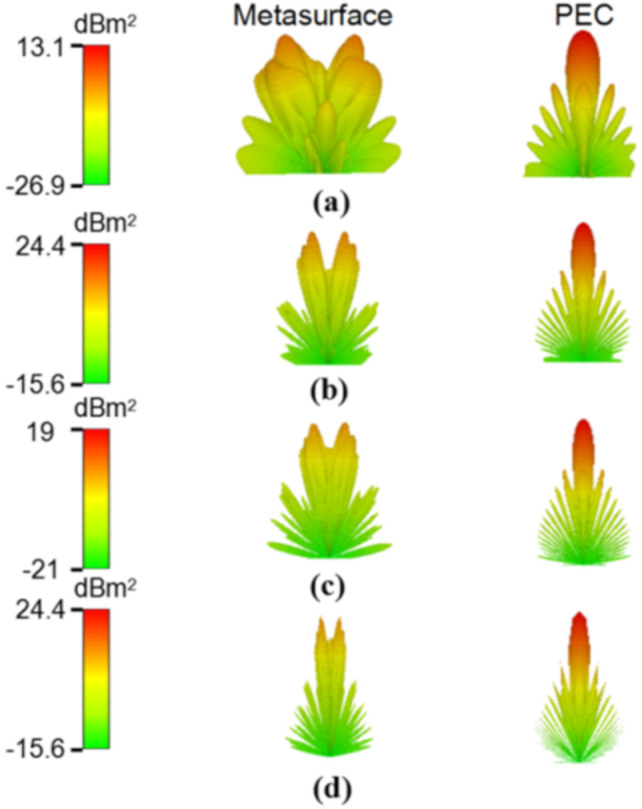
Three-dimensional scattering patterns of metasurface and PEC at different frequencies: (**a**) 7.2 GHz, (**b**) 14.6 GHz, (**c**) 17.2 GHz, and (**d**) 27.6 GHz.

**Figure 9:**
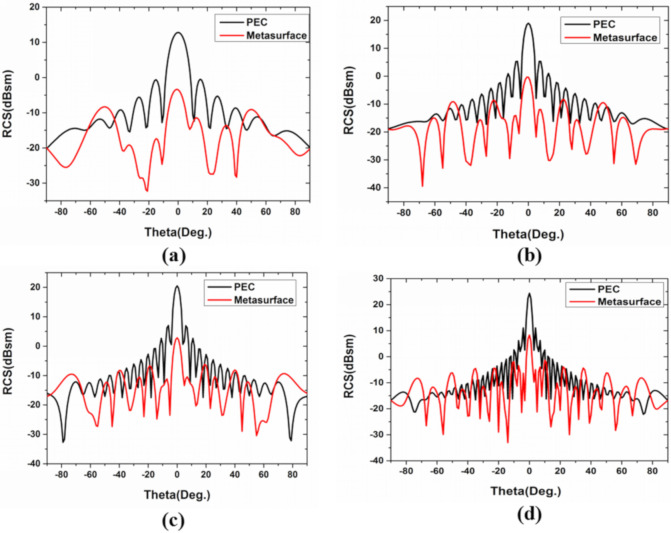
Two-dimensional scattering patterns for (**a**) 7.2 GHz, (**b**) 14.6 GHz, (**c**) 17.2 GHz, and (**d**) 27.6 GHz of metasurface and PEC surface.

To further confirm the diffuse scattering reduction of the metasurface, the three-dimensional and two-dimensional far-field simulation is performed at different frequencies (7.2, 14.6, 27.6, and 29.6 GHz), under normal incidence, with 0° polarization, as shown in Figure 8 and Figure 9. These four frequencies are selected for analysis because the phase difference corresponding to their unit cells is 180 degrees. It can be seen from the comparison chart of the far-field RCS reduction that the energy in the normal direction of the metasurface is greatly reduced compared with equal-sized PEC, and the highest energy of metasurface is also much lower than that of PEC surface in Figure 8. In addition, compared with the PEC surface, the reflected mode of the metasurface is multidirectional scattering. It is noteworthy that the effective periodicity of the AF decreases as the frequency increases, leading to close concentrations of the scattering energy. Finally, it can be seen that the normal reflection energy of the metasurface is 10 dB larger than that of PEC from the two-dimensional scattering patterns of the metasurface and PEC surface in Figure 9.

**Figure 10:**
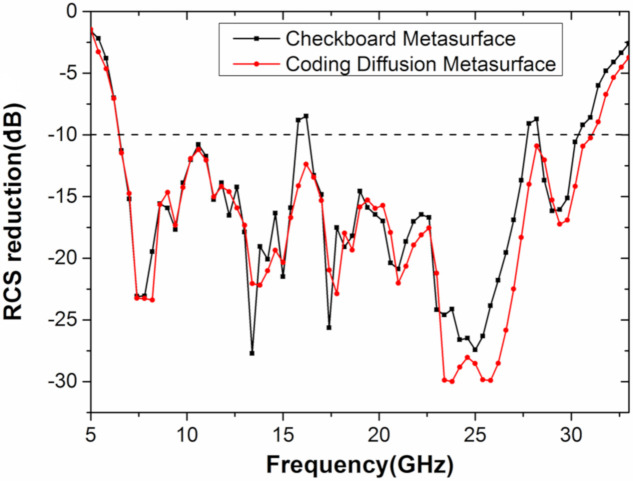
The monostatic RCS reduction of a checkerboard metasurface and a coding diffuse metasurface.

What is more, the optimal design structure in this paper has greater advantages over the traditional checkerboard design. Firstly, the bandwidth corresponding to the checkerboard structure is not, as well as the digital metasurface in this article, and the RCS reduction of the checkerboard structure at some frequencies has not been decreased by 10 dB, as shown in Figure 10. Secondly, the checkerboard structure divides the normal energy into four beams, and the digital metasurface in this paper divides the energy into eight beams, which not only reduces the normal energy but also further reduces the side lobe energy. Therefore, the optimal structure is not a checkerboard structure but an optimized metasurface.

## 4. Fabrication and Measurement

**Figure 11:**
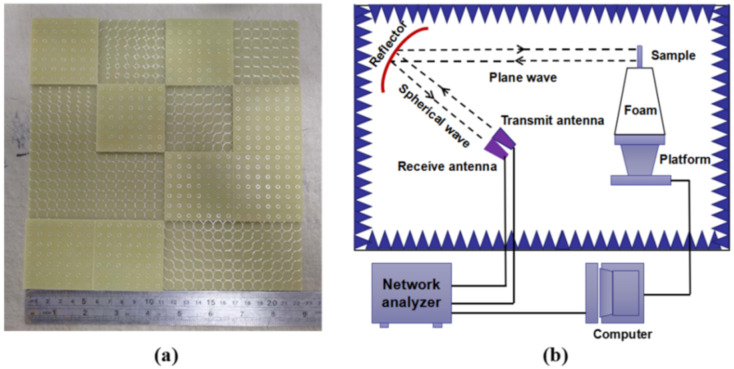
The fabricated metamaterial sample and test schematic: (**a**) the sample and (**b**) the schematic view of a compact range system for measurement.

To verify the abovementioned theoretical design and simulation, a proposed metasurface with a size of 224 mm × 224 mm is fabricated. The dielectric material is FR4 Epoxy Glass Cloth with a dielectric constant of *ε_r_* = 4.3 (loss tangent *tan δ* = 0.022) and the copper thickness on the patch and ground is 35 mm. The sample is fabricated by traditional printed circuit board (PCB) printing technology, which is formed by pressing two boards with the same thickness. The monostatic and bistatic RCS reduction of the sample are measured by the compact range system of the National Electromagnetic Scattering Laboratory in Beijing. As shown in Figure 11, the sample is placed in a microwave anechoic chamber, and the compact range system consists of a set of receiving antennas and transmitting antennas that contain three broadband antennas working in the frequency ranges of 2–18, 18–26.5, and 26.5–40 GHz. A spherical wave is transformed into a plane wave after passing through the reflector, and then the received signal is passed through the vector network analyzer, to obtain the data. Such far-field experimental conditions can be easily obtained by the design between the sample and the reflector that it is in line with the actual working principle of radar.

**Figure 12:**
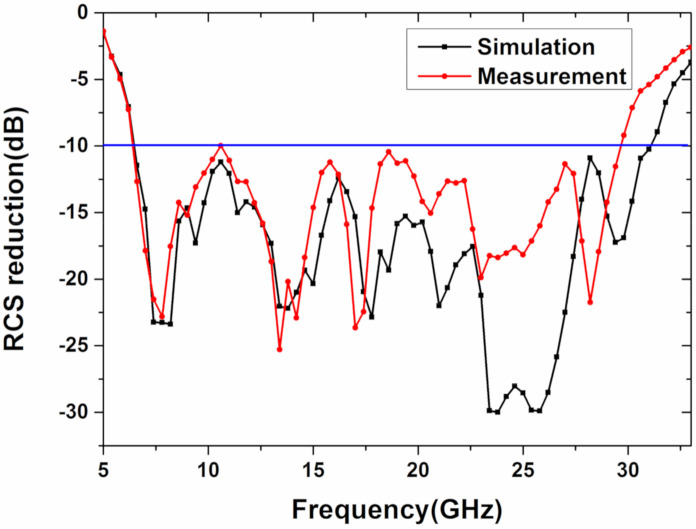
The simulation and measurement of the monostatic RCS reduction, under the x-polarized normal incident.

**Figure 13:**
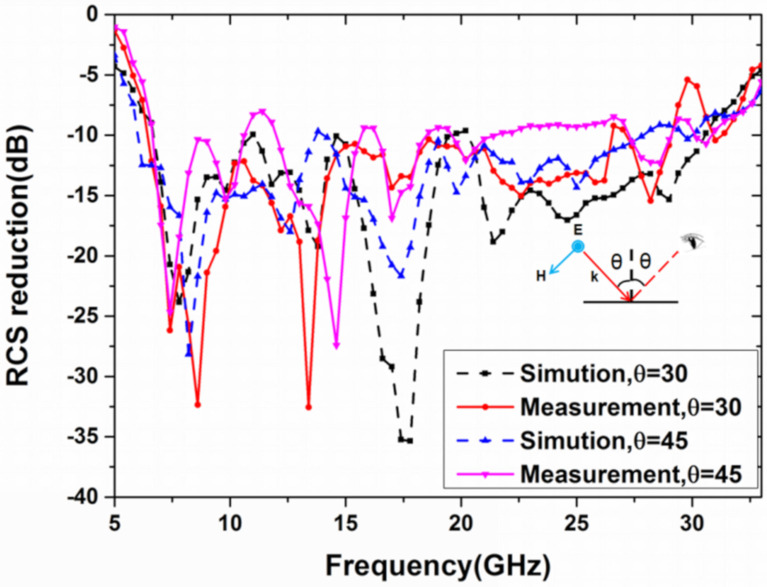
The simulation and measurement of the bistatic RCS reduction under the x-polarized in the incident angle *θ* = 30° and 45°.

The reflection coefficient of the sample can be acquired by the vector network analyzer, and the results are shown in Figure 12 and Figure 13. The monostatic RCS reduction of the metasurface is larger than 10 dB, in the frequency range of 6.4–29.6 GHz, in Figure 12. Moreover, its bandwidth ratio is 4.62:1 (129% of relative bandwidth). A comparison between simulation and experiment under the oblique incidence of 30 and 45 degrees in x polarization is also demonstrated in Figure 13. Although the bistatic RCS reduction of the metasurface is lower than 10 dB in some frequency points under the oblique incidence of 45 degrees, the metasurface shows angle insensitive overall. Some deviation occurs between the experimental results and simulation due to the fabrication and measurement error. Then, Table 1 shows the comparison of our work and the previously reported results. It can be seen that the non-planar structure is beneficial to further broaden the bandwidth, compared to the planar structure, and the bandwidth in this paper is greatly improved through the non-planar structure, compared to the reported work mentioned in the literature. What is more, the metasurface in this paper is polarization-insensitive and angle-insensitive from the simulation and experiment, which is not mentioned in References [20,21]. Thus, through theoretical design, simulation, and experimental verification, we have successfully designed an ultra-wideband uneven metasurface with coding diffuse scattering function.

## 5. Conclusions

In this paper, a novel uneven metasurface is designed and demonstrated for wideband RCS reduction and diffuse scattering. The proposed metasurface is composed of two circle ring structures with different layer thickness that introduces a height variable parameter for phase differences to obtain super-wideband manipulation of EM waves. It features ultra-bandwidth and polarization and angle insensitivity in simulation and experiment, thanks to the inherently isotropic characteristics of the circle ring structure. Through the DPSO algorithm for metasurface, the scattering direction of EM waves becomes more diverse, compared with metal surfaces and checkerboard surfaces. The 10 dB RCS reduction frequency range of the metasurface is from 6.4 to 29.6 GHz, which occupies 129% of the total bandwidth. The theoretical analysis, simulation, and experiment results are perfectly consistent and confirm that the metasurface has a wider RCS reduction and diffuse scattering effect. This work has a great commercial prospect in the RCS reduction and other microwave applications.

## Figures and Tables

**Table 1:** Comparison of this work and previous researches.

Article	RCS Reduction (dB)	Frequency Range (GHz)	Relative Bandwidth (%)	Ratio Bandwidth (f_H_/f_L_)	Planar or Non-Planar
12	10	14.5–21.8	41	1.5	planar
13	10	9.3–15.5	50	1.67	planar
14	10	8.8–17.3	65.2	1.97	planar
15	10	4.10–7.59	60	1.85	planar
16	9.5	8.6–22.5	92	2.62	planar
17	10	5.9–12.2	70	2.07	planar
22	10	17–42	84.75	2.47	planar
23	10	7.9–20.8	89.9	2.63	planar
24	10	15.6–36.5	80.2	2.34	planar
27	10	5.8–18	105.2	3.10	non-planar
28	10	6.2–25.7	122	4.22	non-planar
This work	10	6.4–29.6	129	4.62	Non-planar
